# Primary care screening for peripheral arterial disease: a cross-sectional observational study

**DOI:** 10.3399/bjgp17X689137

**Published:** 2017-01-27

**Authors:** Jane H Davies, Jonathan Richards, Kevin Conway, Joyce E Kenkre, Jane EA Lewis, E Mark Williams

**Affiliations:** South East Wales Trials Unit (SEWTU), Centre for Trials Research, Cardiff University, Cardiff.; Cwm Taf University Health Board, Merthyr Tydfil.; Cwm Taf University Health Board, Merthyr Tydfil.; Faculty of Life Sciences and Education, University of South Wales, Pontypridd.; School of Health Sciences, Cardiff Metropolitan University, Cardiff.; Faculty of Life Sciences and Education, University of South Wales, Pontypridd.

**Keywords:** cardiovascular risk assessment, general practice, peripheral arterial disease, primary health care, screening

## Abstract

**Background:**

Early identification of peripheral arterial disease (PAD) and subsequent instigation of risk modification strategies could minimise disease progression and reduce overall risk of cardiovascular (CV) mortality. However, the feasibility and value of primary care PAD screening is uncertain.

**Aim:**

This study (the PIPETTE study — Peripheral arterial disease In Primary carE: Targeted screening and subsequenT managEment) aimed to determine the value of a proposed primary care PAD screening strategy. Outcomes assessed were: prevalence of PAD and agreement of ankle– brachial index (ABI)-defined PAD (ABI ≤0.9) with QRISK^®^2-defined high CV risk (≥20).

**Design and setting:**

A cross-sectional observational study was undertaken in a large general practice in Merthyr Tydfil, Wales.

**Method:**

In total, 1101 individuals with ≥2 pre-identified CV risk factors but no known CV disease or diabetes were invited to participate. Participants underwent ABI measurement and QRISK2 assessment, and completed Edinburgh Claudication Questionnaires.

**Results:**

A total of 368 people participated in the study (participation rate: 33%). Prevalence of PAD was 3% (*n* = 12). The number needed to screen (NNS) to detect one new case of PAD was 31. Refining the study population to those aged ≥50 years with a smoking history reduced the NNS to 14, while still identifying 100% of PAD cases. Of participants with PAD, 33% reported severe lifestyle-limiting symptoms of intermittent claudication that warranted subsequent endovascular intervention, yet had not previously presented to their GP. The QRISK2 score predicted high CV risk in 92% of participants with PAD.

**Conclusion:**

The low PAD yield and the fact that QRISK2 was largely comparable to the ABI in predicting high CV risk suggests that routine PAD screening may be unwarranted. Instead, strategies to improve public awareness of PAD are needed.

## INTRODUCTION

The clinical relevance of peripheral arterial disease (PAD) stems not only from its well-known debilitating symptoms and sequelae (such as intermittent claudication, ischaemic rest pain, and limb amputation) but also from its position as a strong predictor of future cardiovascular (CV) events. PAD is a marker of systemic atherosclerosis; regardless of whether it is symptomatic or not, it has been repeatedly associated with a three- to six-fold increased risk of death from CV causes.^1^ Furthermore, this increased risk is independent of, and in addition to, that expected by concomitant traditional CV risk factors.^2^ The evidence is sufficiently robust that national and international guidelines recommend the same strategy of CV risk modification for PAD as for coronary artery disease.^3^^–^5 The disease, however, is underdiagnosed and this may be partly attributed to the fact that up to two-thirds of patients with PAD in the community are asymptomatic.^4^ This has resulted in calls for the instigation of primary care PAD screening via ankle–brachial index (ABI) measurement.

The ABI is a measure of the ankle systolic blood pressure relative to central aortic blood pressure (approximated by measuring the brachial systolic pressure). An ABI of ≤0.9 is considered diagnostic of PAD, a cutoff point that has been shown to be >95% sensitive in detecting angiogram-positive disease and approximately 99% specific in identifying healthy subjects.^6^

Studies have demonstrated that an abnormal ABI (≤0.9 or >1.3) is highly prevalent among individuals not considered at high risk of CV events, as defined by CV risk scoring systems such as the Framingham Risk Score (FRS).^7^ According to Grøndal and Lindholt, nearly 25% of CV deaths occur in individuals believed to have low CV risk according to traditional risk stratification models;^8^ this has resulted in suggestions that the ABI, as a non-invasive and inexpensive test, could be added as an additional risk parameter to CV risk tools and/or algorithms.^1^

Current perspectives of PAD screening in the UK appear to be mixed: although UK general practices are awarded Quality and Outcomes Framework (QOF) points for having a register of patients with PAD and for meeting PAD-related targets, there is no incentive to screen patients without symptoms of the disease. Some countries (for example, the Netherlands and Australia) now offer remuneration for ABI measurement in primary care, but this is not the case in the UK. Notably, however, the UK National Screening Committee’s handbook for vascular risk assessment, risk reduction, and risk management refers to the ABI within a list of emerging novel risk factors, but reports that there is insufficient evidence to justify its inclusion in risk assessment scores at present.^9^ The concept of early identification of CV risk factors and disease emerges as a pivotal theme in the UK Department of Health’s *Cardiovascular Disease Outcomes Strategy*, with particular reference to PAD.^10^

**How this fits in**

Routine PAD screening and subsequent appropriate treatment could minimise progression of the disease and reduce overall cardiovascular (CV) risk. This study has shown that targeting individuals aged ≥50 years who have a history of smoking could be an effective and efficient PAD screening strategy; however, results also suggest that QRISK2 could be a more amenable and comparable alternative for the identification of high CV risk in the primary care setting.

This study (the PIPETTE study — Peripheral arterial disease In Primary carE: Targeted screening and subsequenT managEment) aimed to determine the value of a proposed primary care PAD screening strategy. The outcomes assessed were:

- prevalence of undiagnosed PAD/yield from screening; and
- agreement of ABI-defined PAD (ABI ≤0.9) with a QRISK^®^2-defined high CV risk (score ≥20).

## METHOD

This prospective observational study was based in a large general practice (11 426 patients) in Merthyr Tydfil, Wales. This is an area with high levels of deprivation, adverse health behaviours, and morbidity.^11^

The study population was chosen on the premise that screening should allow the identification of patients who would stand to benefit from any PAD diagnosis (defined as ABI ≤0.9 for one or both legs) and subsequent treatment; those with diabetes or known cardiovascular disease (CVD) were excluded as they should have already been targeted for secondary preventive strategies. Full inclusion and exclusion criteria are outlined in Box 1.

Box 1. **Study inclusion and exclusion criteria**

**Table:** 

Inclusion criteria Males aged ≥45 years or females aged ≥55 years (age-related CVD risk factor); At least one *additional* CVD risk factor from the following: – cigarette smoking or regular exposure to passive smoke (that is, living with a smoker); – hypertension (systolic blood pressure of ≥140 mmHg, diastolic blood pressure of ≥90 mmHg, or taking antihypertensives); – Low high-density lipoproteins (<1.0 mmol/L), high low-density lipoproteins (>3.3 mmol/L), high triglycerides (>1.7 mmol/L), or taking lipid-lowering medication; – family history of premature coronary heart disease (first-degree male relative aged <55 years, first-degree female relative aged <65 years); – elevated waist circumference (≥102 cm in non-Asian males, ≥90 cm in Asian males, ≥88 cm in non-Asian females, ≥80 cm in Asian females); – BMI of >25; Willingness to participate in the study. Exclusion criteria Diabetes mellitus (type 1 or 2); Known coronary heart disease, including history of myocardial infarction, angina (stable or unstable), coronary artery procedures (coronary artery bypass graft or percutaneous coronary intervention), or evidence of clinically significant myocardial ischaemia; Known cerebrovascular disease (for example, history of transient ischaemic attack or stroke); Known peripheral arterial disease; Known non-coronary forms of atherosclerotic disease (for example, abdominal aortic aneurysm); Serious or unstable medical or psychological conditions that, in the opinion of the investigator or patient’s GP, would compromise the patient’s safety or successful participation in the study; Current or recent (preceding 4 months) participation in a clinical research trial (this does not apply to participation in non-interventional research); Patient who is unwilling or unable to provide informed consent.

BMI = body mass index. CVD = cardiovascular disease.

**Figure 1.**
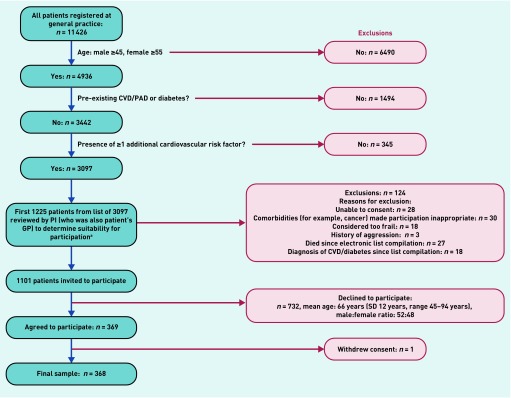
***Recruitment process.*** ***^a^Time and financial limitations of the study prevented invitation and consideration of full list of 3097 patients. CVD = cardiovascular disease. PAD = peripheral arterial disease. PI = primary investigator. SD = standard deviation.***

Figure 1 outlines the recruitment process. In total, 1101 individuals with ≥2 pre-identified CV risk factors (the first of these was always age related, that is, male ≥45 years, female ≥55 years) but no known CVD (including known PAD) or diabetes mellitus were identified via a search of the practice’s electronic patient database and invited by letter to participate. No attempt was made to contact non-responders. Written, informed consent was gained from each participant.

Difficulty recruiting to health research in primary care is well documented and can be partly attributed to logistical factors such as domestic and/or work issues, illness, and transport.^12^ Furthermore, attendance to screening declines with age; conversely, PAD incidence increases with age.^8^ This study aimed to address these issues by offering participants the option of being seen at home or at their medical practice.

Participant medical records were reviewed for past medical history and current medications. Participants underwent physical examination — the components of which are detailed in Box 2 — and were assisted by the research nurse to complete the Edinburgh Claudication Questionnaire (ECQ), which is an established, validated, intermittent claudication diagnosis tool.^13^ All measurements, including ABIs, were undertaken by the research nurse. Participants were asked about their current smoking status and alcohol intake. A venous blood sample was obtained from those who were found to have PAD following 12 hours of fasting, and analysed to determine the fasting glucose level and lipid profile. The QRISK2 CV risk algorithm was used to calculate a 10-year CV risk score for each participant. For those participants who did not have a blood sample taken (no PAD), or for whom data were missing, the most recent data in their medical record were used to calculate their QRISK2 score.

Box 2. **Components of physical assessment**

**Table:** 

Height: without shoes, measured in metres using a Seca Leicester Portable stadiometer; Weight: without outer clothes and shoes, measured in kilograms using Seca 877 floor scales for mobile use (class III); Waist circumference: undertaken according to the World Health Organization’s data-gathering protocol;^14^ Hip circumference: undertaken according to the World Health Organization’s data-gathering protocol;^14^ Blood pressure: measured using a Welch Allyn^®^ aneroid sphygmomanometer and stethoscope, in accordance with British Hypertension Society guidelines for blood pressure measurement;^15^ Pulse: by palpating the radial pulse and counting the number of pulses for a 1-minute period; Assessment for clinical signs of PAD: reduced or absent pulses in legs/feet, thickened nails, smooth shiny skin, hair loss to legs/feet, pallor or cyanosis to legs/feet, pallor on elevation of legs, legs/feet appearing flushed in a dependent position, reduced temperature to one or both legs/feet; ABI measurement: according to the American Heart Association scientific statement.^16^

ABI = ankle–brachial index. PAD = peripheral arterial disease.

### Statistical analysis

Data analysis was performed using statistical software SPSS (version 21). Categorical data were assessed using the χ^2^ test or Fisher’s exact test. Continuous data were assessed using an independent *t*-test or Mann–Whitney *U* test (as determined by the Shapiro–Wilk test of normality). Significance was set at *P*<0.05.

## RESULTS

Data from 368 out of a possible 1101 participants were collected, giving a participation rate of 33%. Most participants (63%) chose to be seen at home; the remaining 37% were seen at the medical practice. Population characteristics and physical assessment results are presented in Table 1.

**Table 1.** Population characteristics and physical assessment results

	**PAD^b^*n*= 12**	**No PAD^c^*n*= 356**	**All *n*= 368**	***P*-value**
Age, years^a^	70.6 (±9.6) 56–86	63.6 (±8.2) 45–85	63.8 (±8.3) 45–86	0.02^d^

Male:female sex ratio	58:42	54:46	55:45	0.54^e^

White British ethnicity, %	100	100	100	NA

Smoking status, *n* (%)				<0.001^f^
Current smoker	6 (50)	37 (10)	43 (12)	
Ex-smoker	6 (50)	125 (35)	131 (36)	
Non-smoker	0 (0)	194 (54)	194 (53)	

Family history of premature CHD, *n* (%)	2 (17)	94 (26)	96 (26)	0.53^f^

Systolic BP, mmHg	144 (±10) 130–160	140 (±16) 98–198	140 (±16) 98–198	0.18^d^

Diastolic blood pressure, mmHg	76 (±13) 54–98	81 (±9) 40–113	81 (±10) 40–113	0.15^d^

Hypertension, defined as raised systolic and/or raised diastolic BP and/or on medication for hypertension, *n* (%)	10 (83)	268 (75)	278 (76)	0.1^e^

Pulse pressure, mmHg	68 (±10) 52–88	59 (±14) 30–106	59 (±14) 30–106	0.008^d^

Heart rate, beats per minute	80 (±16) 58–103	74 (±12) 40–114	74 (±12) 40–114	0.095^g^

Dyslipidaemia, *n* (%)				
Yes	11 (92)	246 (69)	257 (70)	0.23^e^
No	1 (8)	74 (21)	75 (21)	
No data available	0 (0)	36 (10)	36 (9)	

Triglycerides >150 mg/dL or 1.7 mmol/L, *n* (%)	3 (25)	116 (33)	119 (32)	0.55^f^

HDL <40 mg/dL or 1.0 mmol/L, *n* (%)	0 (0)	35 (10)	35 (10)	0.62^f^

LDL ≥130 mg/dL or ≥3.3 mmol/L, *n* (%)	3 (25)	137 (38)	140 (38)	0.22^e^

Taking lipid-lowering medication, *n* (%)	7 (58)	76 (21)	83 (23)	0.03^f^

BMI, kg/m^2^	27 (±4) 22–37	30 (±5) 19–54	29 (±5) 19–54	0.07^d^

Waist circumference, cm	98 (±11) 83–114	100 (±14) 64–143	100 (±14) 64–143	0.566^d^

Total number of CV risk factors	4 (±1) 3–5	3 (±1) 1–5	3 (±1) 1–5	0.016^g^

Chronic kidney disease, *n* (%)	0 (0)	11 (3)	11 (3)	1.0^f^

Atrial fibrillation, *n* (%)	1 (8)	10 (3)	11 (3)	0.29^f^

Rheumatoid arthritis, *n* (%)	2 (17)	5 (1)	7 (2)	0.019^f^

QRISK^®^2 score	32 (±12) 11–59	18 (±10) 3–58	19 (±11) 3–58	0.001^d^

Relative risk according to QRISK^®^2	1.6 (±0.6) 1.1–2.9	1.3 (±0.6) 0.6–5.6	1.3 (±0.5) 0.6–5.6	0.016^d^

≥1 clinical sign(s) of PAD, *n* (%)	9 (75)	64 (18)	73 (20)	<0.01^e^

Positive ECQ score, *n* (%)	5 (42)	6 (2)	11 (3)	<0.01^e^

a Unless otherwise stated, data are presented as mean, standard deviation, range.

b *ABI* ≤*0.9.*

c *ABI >0.9*.

d *Mann–Whitney* U *test.*

e *χ^2^ test*.

f *Fisher’s exact test*.

g t*-test.*

ABI = ankle–brachial index. BMI = body mass index. BP = blood pressure. CHD = coronary heart disease. CV = cardiovascular. ECQ = Edinburgh Claudication Questionnaire. HDL = high-density lipoprotein. LDL = low-density lipoprotein. PAD = peripheral arterial disease.

The prevalence of PAD within the study population was 3% (*n* = 12). Of these, 42% (*n* = 5) reported symptoms of intermittent claudication and had a positive ECQ result; 80% of those five individuals (*n* = 4) reported severe lifestyle-limiting intermittent claudication that warranted referral to a vascular surgeon and subsequent endovascular intervention (angioplasty). None of these participants had previously reported their symptoms to their GP as they regarded them as a ‘normal part of ageing’ or a sign of a lack of physical fitness.

Factors found to be significantly associated with PAD included:

- advancing age (*P* = 0.02);
- current smoking (*P*<0.01);
- pulse pressure (*P*<0.01);
- rheumatoid arthritis (*P* = 0.019);
- QRISK2 score (*P*<0.01)
- positive ECQ result (*P*<0.01); and
- the presence of ≥1 clinical sign of PAD (*P*<0.01).

The number needed to screen (NNS) to detect one new case of PAD was 31. Refining the study population to those aged ≥50 years with a history of smoking (ex- or current smoker), based on factors found to be significantly associated with PAD, would have reduced the NNS to 14, while still identifying 100% of the individuals with PAD.

**Figure 2.**
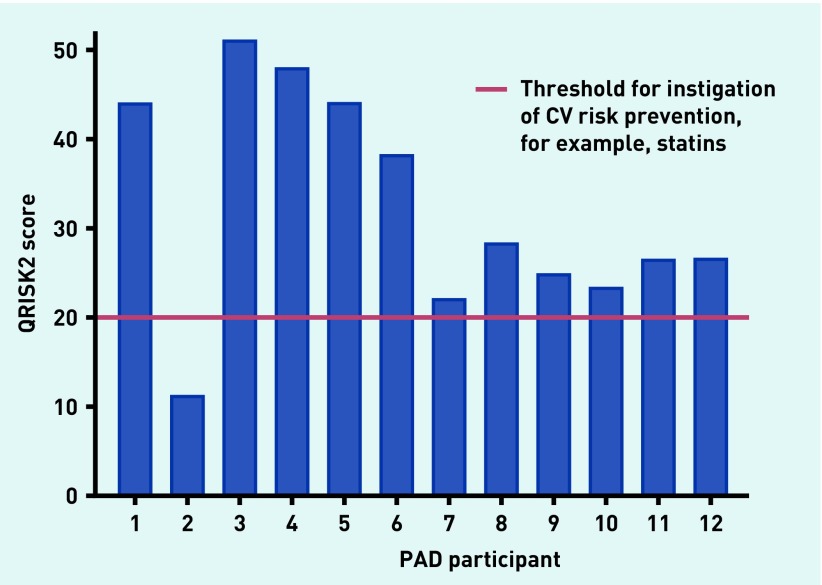
***QRISK2 score for participants with PAD. CV = cardiovascular. PAD = peripheral arterial disease.***

The QRISK2 score predicted a high CV risk (defined by a QRISK2 score of ≥20) in 92% (*n* = 11) of participants with PAD (Figure 2).

## DISCUSSION

### Summary

Although, globally, there is no shortage of studies investigating the prevalence of PAD, this PIPETTE study is the first UK study of PAD prevalence undertaken for 9 years.^17^^–^19 The attained prevalence rate of 3% was lower than anticipated, given the social and health demographics of the area in which the study was undertaken. A targeted screening population, aged ≥50 years with a history of smoking, would provide an effective and efficient screening strategy (NNS = 14), which also concurs with recommendations from the American College of Cardiology Foundation (ACCF) and the American Heart Association (AHA).^3^ However, as QRISK2 predicted high CV risk in 92% of participants with PAD, it appears that the effect of adding the ABI to the QRISK2 algorithm, as an additional risk parameter, would be minimal.

A third of participants with PAD had severe symptoms of intermittent claudication that warranted subsequent endovascular intervention, but had not presented to a doctor. This fact suggests a lack of public awareness of the disease and its symptoms.

### Strengths and limitations

This single-centre study with a relatively small sample size reduces the generalisability of the findings. In addition, all participants were of white British origin and, as such, are unrepresentative of the UK population. This lack of ethnic diversity could, in part, be due to the fact that only 3% of the population of Merthyr Tydfil (compared with 19.5% for England and Wales) is of non-white and non-British origin.^20^^,^^21^ Furthermore, this could have affected results as rates of CVD are known to be higher in people of South Asian and African Caribbean origin, for example.^22^

One aspect of the design of this study — home participation — could be considered both a strength and a limitation. Allowing participants to take part at home could have increased the recruitment of those with mobility problems, transport issues, or caring duties for family members. This could have resulted in improved epidemiological data in terms of acquiring PAD prevalence rates that are more accurate. Conversely, however, incorporating home visits into the study design could have meant that the resultant study population was not representative of those individuals who would ordinarily be likely to come forward for health service PAD screening undertaken in a healthcare setting. This could mean the results may not be transferable or applicable to an actual screening programme. Furthermore, recruitment bias, with individuals who are more health conscious being more likely to agree to take part than those who are less health conscious, was also possible.

Despite its limitations, this study serves to add to the evidence base regarding the epidemiology of PAD in the UK and globally.

### Comparison with existing literature

#### PAD prevalence

Comparing the attained PAD prevalence rate of 3% with existing data is hindered by the fact that studies use different methods for calculating the ABI. Although several studies screened similar populations as this PIPETTE study in terms of excluding those with pre-identified CVD,^22^^–^25 only two (the Multi-Ethnic Study of Atherosclerosis [MESA] and PANDORA studies^25^^,^^26^) calculated the ABI using the standard calculation as recommended by the AHA.^16^

In the US, the MESA study, which screened 1932 individuals aged 45–84 years, who were free of known clinical CVD, returned a similar PAD prevalence of 3.4%.^25^ However, the pan-European PANDORA study of 9816 individuals, with no known CVD or diabetes, reported a higher prevalence of 17.8%.^26^ The PANDORA study differed from the MESA study in that its inclusion criteria specified that participants must have ≥2 CV risk factors, which may account for some of the discrepancy in prevalence rates between those two studies. The PIPETTE prevalence rate is much closer to that of the MESA study, despite the fact that its inclusion and exclusion criteria are almost identical to those of the PANDORA study. Prevalence rates of British studies range from 8.0–10.8% but, again, differing study populations and ABI calculation methods make comparisons difficult.^17^^–^19

#### Who should PAD screening target?

The suggested PAD screening target population of people aged ≥50 years with a history of smoking concurs with that recommended by the ACCF/AHA Task Force’s PAD guidelines.^3^ Adhering to the UK PAD guideline formulated by the National Institute for Health and Care Excellence — which recommends screening people with diabetes and those with symptoms of PAD or non-healing leg wounds — would have resulted in an NNS of 3, with only 42% of PAD cases being identified.^5^

In comparison with existing screening programmes in the UK, the proposed screening target population for PAD would be more efficient in terms of the yield of positive cases. The NNS to detect one new case of PAD (NNS = 14) is less than the reported NNS to detect one positive case associated with current bowel screening (NNS = 50)^27^ and breast screening (NNS = 125)^28^ programmes. Though, of course, the true value of a screening programme is better assessed via consideration of the NNS for a given duration to prevent one death or adverse event. That information is derived from randomised control trials (RCTs) of screening versus no screening, of which there is none currently published in relation to PAD. It should be noted, however, that data from the Viborg Vascular PAD screening RCT, including all-cause and CVD mortality, should be available in late 2018.^29^

#### Cardiovascular risk prediction

Some studies have questioned the value of adding the ABI to CV risk algorithms.^7^ Murphy *et al* examined the predictive ability of ABI compared with the FRS by conducting a post-hoc analysis of data from the ARIC (Atherosclerosis Risk in Communities) Study;^30^ they concluded that the independent effect of ABI when adjusted for the FRS was small and did not support FRS modification to include ABI. Newer CV risk scoring systems such as the Joint British Societies’ consensus (JBS3) and QRISK2 are considered improvements on the FRS as a result of their incorporation of additional risk factors such as ethnicity, family history, and social deprivation; notably, however, no studies to date have assessed the contribution of the ABI to these superior CV scoring tools.^31^ The UK National Screening Committee highlights that a major drawback of CV risk assessment algorithms concerns missing or out-of-date data, which may reduce their accuracy and undermine confidence in predictive ability.^9^

#### Lack of public awareness of PAD

Existing studies have also reported a lack of public awareness of PAD; according to Norgren *et al*, population studies have consistently shown that 10–50% of patients with intermittent claudication have never consulted a doctor.^32^ Studies by Hirsch *et al* and Lovell *et al*, conducted in the US and Canada respectively, demonstrated that approximately two-thirds of people surveyed had never heard of PAD;^33^^,^^34^ in Hirsch *et al*’s study, of those who were aware of it, half or fewer were unaware that smoking (44%) and diabetes (50%) could lead to PAD.^33^ Results from the PIPETTE study appear to suggest that this apparent lack of awareness of PAD also applies in the UK and has not improved in recent years.

### Implications for research and practice

This PIPETTE study provides evidence that routine primary care PAD screening in a non-diabetic population is not worthwhile. However, larger-scale studies that incorporate a population derived from multiple general practices and that is ethnically diverse are required to corroborate results.

With regard to identification of individuals at high CV risk, it appears that the QRISK2 algorithm is largely comparable to the ABI, with the former being far more amenable for use in busy general practice settings. As most CV risk algorithms are now incorporated into general practice electronic health record systems, with new information, such as an updated blood pressure, being automatically processed to continually update the risk score, a health professional can determine a patient’s score at the touch of a button. In contrast, ABI measurement can be impaired by issues relating to its practicality and the requisite operator skill at using the handheld Doppler.^35^

This study suggests that there is room for improvement in the primary care diagnosis of symptomatic PAD. This could be achieved by clinicians simply asking about claudication symptoms during routine consultations. Effective strategies to raise public awareness of this little known disease are also needed. On a simplistic level, this could involve displaying PAD posters in general practice waiting rooms and pharmacies; more sophisticated strategies, via social media, for example, could also be developed and implemented.
